# The dual role of academic surgeons as clinicians and researchers - an attempt to square the circle?

**DOI:** 10.1186/1754-9493-5-16

**Published:** 2011-06-22

**Authors:** Markus Huber-Lang, Edmund Neugebauer

**Affiliations:** 1 Department of Traumatology, Hand-, Plastic- and Reconstructive Surgery, University Hospital of Ulm, Steinhövelstraße 9, D-89075 Ulm, Germany; 2 Institute for Research in Operative Medicine (IFOM), University Witten/Herdecke, Campus Cologne, Ostmerheimer Str. 200, Haus 38, D-51109 Köln, Germany

## Academic research - a prerequisite for patient safety in surgery

In the past experimental and clinical research efforts in surgery have been considered the "golden key" for understanding the underlying pathomechanisms of surgical diseases, for successful development of surgical techniques, improved patients quality of life and beneficial clinical outcome. Furthermore, scientific findings and evidence based behaviour represented the basis for the management of patients undergoing surgical procedures accompanied with an increased patient's safety in surgery.

However, in our days of "limited time and financial resources", combining the clinical and research challenges on a necessary high level of quality seems to be more and more challenging, possibly jeopardizing patient's safety in the future.

## Stating the Problem

During the middle ages a large discrepancy already existed in terms of content and personnel between the profession of surgery (*from old french: "serurgien, cirurgien", from latin: "chirurgia" = "working or done by hand") *and research *(from old french "recercher" = "seek out, search closely"*), and accordingly science (*scientia = "knowledge"*). Currently, both clinicians and researchers in daily routine often also question the compatibility of surgery and research, and sometimes describe this as a "squaring of the circle", in the sense of a metaphor that is unsolvable. If in particular the young surgical researcher is given the impression that this is a realistic picture, or has in reality scarcely experienced state-of-the-art clinics combined with profound research efforts, they will already be discouraged at an early stage from long-term research. Consequently, young surgeons will almost completely concentrate on their clinical performance and training. Furthermore, those who are interested in surgical research experience an increasing division between clinically relevant hypotheses generated from daily surgical care and the rapidly emerging more and more complex scientific methodology. Occasionally, it is even observed at a linguistic level that there is a big difference between "surgery and research". Therefore, the question inevitably arises, whether our interdisciplinary and internationally closely-networked world would also in the future need a "research surgeon" and/or a "surgical researcher"?

## The Necessity for Research in the Training of Young Surgeons

**Figure 1:**
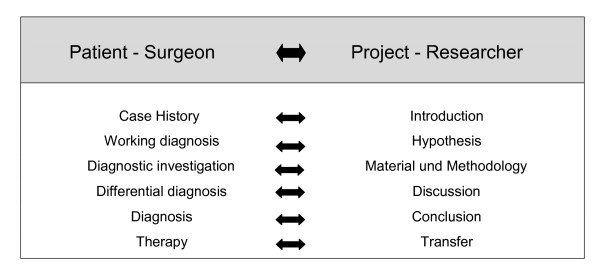
**Translation with regards to the constituents of "surgery and research" during the training phase**.

Getting to the root of a clinical training focussed on patients, which envisages a complex situation for the surgeon with obvious or masked symptoms, then the training of certain analytical abilities is indispensable (Figure 1). Thereby, valid working hypotheses must be formulated and these will be verified by specific, economically-rational diagnostic methods. After internal and external (e.g. cooperative) discussion of potential differential diagnoses, the "detection" (that is the pinpointing of an accurate as possible diagnosis) is of critical importance for the patient. The subsequent consideration of differential therapies, based on the current available scientific evidence - with a generally comprehensible transfer of information to the patient in a patient adapted way - is crucial for the finally chosen therapy. The patient - surgeon relationship must be based on trust to the surgeon to ensure long-term satisfactory therapy for both the patient and the surgeon. Precisely these basic abilities for analysis, creation of hypotheses, reflection as well as internal and external critical appraisal will be considerably honed through an early synchronous scientific training. On the other hand, the as "clinical experience" designated knowledge of direct patient contact will clearly modulate and define the scientific questions. Therefore, the coexistence of "surgery and research" appears imperative, especially during the training phase. However, since surgical training is doubtlessly associated with lifelong learning, the necessity for "surgery and research" is shown as being in far excess of the specialist training period.

## The Two Faces of the Roles of Surgery and Research in the Reality

**Figure 2:**
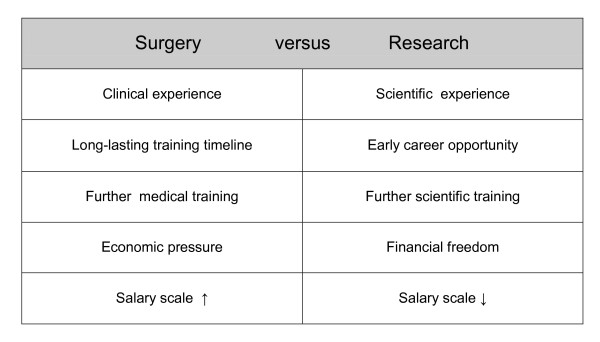
**Antagonisms that can be experienced in "surgery and research" in workaday life**.

The surgeon has at least four main roles - accordingly being depicted as a "square". He must in essence be a doctor with multiple skills, an expert carrying out operations on a manual and technical state-of-the-art, then a critical monitor of his own work during the pre-, intra- and post-operation phase, and furthermore an idealist allowing for the appropriate time frame for all these activities. On the downside there is an increasingly tangible change of the surgeon into a "patient manager" with standard operation procedures, into a specialist with a limited focus, as well as into an economist in an environment with an increasing process optimization and acceleration. In comparison, the main roles assigned to the "researcher" can be accordingly depicted as a "circle" (without start or end). He must possess an inner tireless drive, answer questions, make new discoveries and create new knowledge ("scientist"), which in turn generates new questions. On the downside, the researcher these days transforms himself into a "scientific manager" who carries out "en vogue" and "low-risk research", is subject to increased publication pressure and must spend excessive time on administration and the acquisition of third-party funding. A reconciliation of the outlined fundamental roles of the surgeon and the researcher (including their dark sides) is, therefore, in reality almost impossible, and potentially undesirable. Further generally known, antagonistically presented phenomena of "surgery versus research" are listed in Figure 2. Noteworthy amongst these is above all the incoherence of the to some extent ever still hierarchical structure in surgery with the earlier scientific independence, which is for example stipulated and supported through the modern funding structures of the German Research Foundation (DFG) (junior professors, Emmy Noether Programme, Heisenberg grant, etc., for more details see http://www.dfg.de). Central to this relationship is also the obvious discrepancy between the agreed doctors' salary levels and those of the scientist, which is by far lesser. This partially two-sided reality of "surgery and research" can often be countered for a relative long period by the joy of research and of patient care, and in particular, when both the "research and patient"-centred surgery is lived and experienced. Thereby, direct and indirect, short- and long-term feelings of success arise, which may flow back as motivational energy into the system of "surgery and research". In the long-term it appears, however, that an appropriate appreciation and reward for a clinically and scientifically responsible role is also of importance, especially when this role is accompanied by considerable stress.

## The Complexity of the Life-Work Balance in Surgery and Research

**Figure 3:**
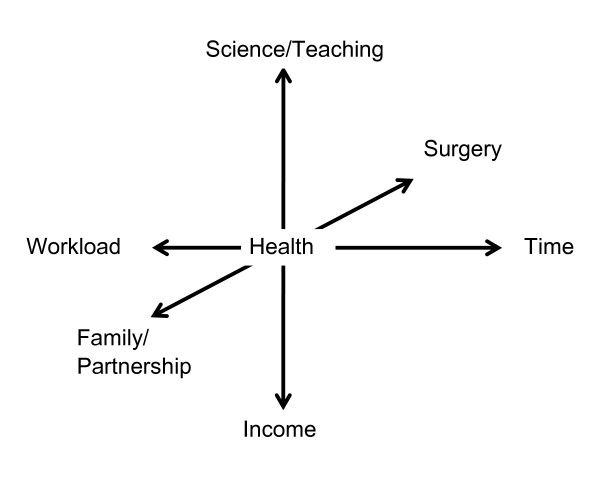
**Complexity of the balance of "work and life" for the research surgeon**.

If one takes the personal health (of body, spirit and soul) of the "research surgeon" or the "surgical researcher", both of whom in their multi-vectorial orientation ideally always have in mind the health of the patient as the main command variable, as the mid-point of the coordinate system (Figure 3), it thus becomes clear, that the complexity and accumulation of the conditions will get to them. The "work-life balance" can readily lose its equilibrium. The result in the medium-term is the threat to their own health as well as to family/partnership and their circle of friends. How can, therefore, this labile coordinate system, whose complexity because of many constant variables cannot be considerably simplified, be lastingly stabilized, which vector values must therefore be strengthened and which weakened?

## The Potential Solutions of the "Squaring of the Circle" of Surgery and Research

**Figure 4:**
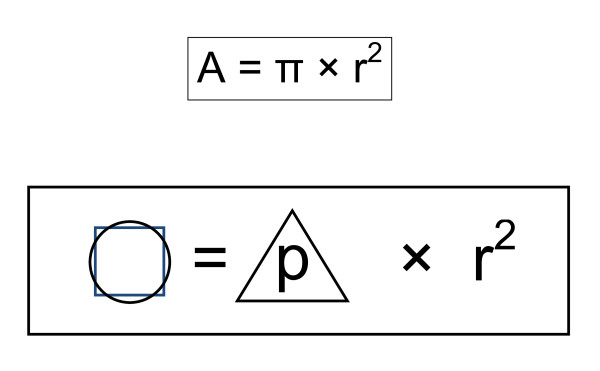
**"Surgery and research": Attempt at "squaring the circle" through the patient-centred triology clinic, research and teaching with a high degree of reliability and room for scientific and personal development**. □ O = "Squaring the circle". Δ = Trilogy: "Patient Care, Science, Teaching". p = "patient-centred". r^2 ^= reliability × room.

**Figure 5:**
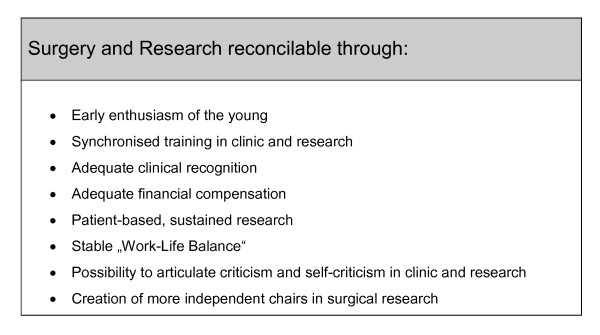
**Important prerequisites for the successful long-term reconciliation of "surgery and research"**.

Historically, the "squaring of the circle" was finally achieved mathematically through the insertion of the *irrational *number π (= 3.14...) within the common area formula A = π × r^2^. In a free association (Figure 4) in the search for a potential solution for the apparently *irrational *reconcilability of "surgery and research", the patient-centred trilogy (= 3) of **research**, **teaching **and **patient care **can be multiplied with a surgical plus scientific **reliability **as well as sufficient **room **in the sense of openness, scope of research, opportunities for development and creation, training and time. This may result in a *rational *convergence on the compatibility of "surgery and research". As a framework for this formula, additional factors are essential, as listed in Figure 5. In particular, early enthusiasm and synchronous training in "surgery and research", adequate clinical recognition and payment, research sustained well above the consultant and postdoctoral levels, articulated criticism and self-criticism in the clinic and research, and an appropriate modern external review structure are required. These are the minimal prerequisites for the reconciliation of "surgery and research", which in the long-term can guarantee and facilitate a stable "work-life balance". Only then it is feasible, that in the future more young doctors and researchers may be recruited to this highly interesting field. As an important pattern for educational measures in this context would be the establishment of new, independent chairs in surgical research closely related to questions from the bedside for the best national and international candidates, which in turn would preferably again result in more numerous "surgical researchers" and "research surgeons".

